# Congenital chloride diarrhoea in a Chinese infant with a compound heterozygous *SLC26A3* mutation

**DOI:** 10.1186/s12887-024-04788-x

**Published:** 2024-05-04

**Authors:** Qian Li, Jing Wang, Ruixian Zang, Lichun Yu, Zhenle Yang, Shuzhen Sun

**Affiliations:** 1 Department of Pediatric Nephrology and Rheumatism and Immunology, Shandong Provincial Hospital, Cheeloo College of Medicine, Shandong University, Jinan, 250021 P.R. China; 2 Department of Pediatric Nephrology and Rheumatism and Immunology, Shandong Provincial Hospital Affiliated to Shandong First Medical University, Jinan, 250021 P.R. China

**Keywords:** Congenital chloride diarrhoea, Solute carrier family 26 member 3, Child, Metabolic alkalosis, Hypochloraemia, Hypokalaemia, And hyponatraemia

## Abstract

**Introduction:**

Congenital chloride diarrhoea (CCD) is an autosomal recessive condition that causes secretory diarrhoea and potentially deadly electrolyte imbalances in infants because of solute carrier family 26 member 3 (*SLC26A3*) gene mutations.

**Case presentation:**

A 7-month-old Chinese infant with a history of maternal polyhydramnios presented with frequent watery diarrhoea, severe dehydration, hypokalaemia, hyponatraemia, failure to thrive, metabolic alkalosis, hyperreninaemia, and hyperaldosteronaemia. Genetic testing revealed a compound heterozygous *SLC26A3* gene mutation in this patient (c.269_270dup and c.2006 C > A). Therapy was administered in the form of oral sodium and potassium chloride supplements, which decreased stool frequency.

**Conclusions:**

CCD should be considered when an infant presents with prolonged diarrhoea during infancy, particularly in the context of maternal polyhydramnios and dilated foetal bowel loops.

## Introduction

Congenital chloride diarrhoea (CCD) is a rare autosomal recessive disease causing severe electrolyte imbalances because of mutation in the intestinal Cl^−^/HCO3^−^ exchanger gene *SLC26A3* (solute carrier family 26 member 3) [1]. During the antenatal period, CCD is commonly characterized by foetal bowel loop dilation, preterm birth, and maternal polyhydramnios and, in some cases, can lead clinicians to suspect antenatal bowel obstruction [2]. After birth, CCD causes severe watery diarrhoea, which can result in negative consequences, including a failure to thrive, metabolic alkalosis, hypochloraemia, hypokalaemia, and hyponatraemia. When not adequately treated, CCD can cause early death due to progressive impairment of renal function, nephrocalcinosis, and consequent end-stage kidney disease, with a subset of CCD patients ultimately undergoing renal transplantation [3, 4]. Accurately diagnosing CCD patients is critical to ensuring that they receive appropriate treatments in a timely manner to improve their long-term prognosis.

CCD has been reported sporadically throughout the world. Finland, Poland and Arab countries are reported to have high incidences of the disease. The incidence of CCD is 1/30 000 ∼ 40 000 in Finland, 1/200 000 in Poland, and 1/3 200 in Kuwait, possibly related to consanguineous mating [5, 6]. However, there have been only a small number of single-centre or multiple-centre studies and case reports examining CCD incidence in East Asia [7, 8], with more than 10 sporadic cases reported in China. It remains to be established whether the clinical or genetic presentations of CCD in East Asian populations differ from those in other populations.

We describe the case of an infant diagnosed with CCD who was found to harbour a compound heterozygous mutation in the *SLC26A3* gene. We explore the clinical and genetic characteristics of Chinese children with CCD in light of this report and related literature.

## Case presentation

The female infant in this report was the second child of healthy parents. During pregnancy, maternal polyhydramnios was detected via ultrasound. The patient was born weighing 2500 g, with a height of 44 cm at 37 weeks gestational age; she had Apgar scores of 10, 10, and 10 at 1, 5, and 10 min after birth, respectively. Beginning on the day of birth, the patient produced 3–6 liquid stools per day and exhibited abdominal distension that did not improve significantly when routine antidiarrhoeal treatment and lactose-free milk were administered. Despite being fed breast milk and cow milk, her weight gain over the first 6 months after birth was very slow, and both her physical growth and motor development were delayed. At 5 months of age, she had learned to steadily raise her head, but she was unable to sit without support and weighed only 6 kg at 7 months of age. There was no family history of protracted diarrhoea during infancy.

The patient was admitted to our hospital with a history of frequent watery diarrhoea (> 10 times per day) and severe dehydration at 7 months of age. Her blood pressure was 84/45 mmHg. Physical examination revealed a dehydrated appearance, depressed spirit, malnutrition, and tachypnoea. No rales were heard in either lung. Her heart rate was 162 beats/min, and her abdomen was distended with active bowel sounds. Haematological analyses revealed a blood haemoglobin level of 11 g/dL, 17.15 µmol/L serum creatinine, 44.6 mmol/L bicarbonate, 131 mmol/L sodium, 2.12 mmol/L potassium, 2.78 mmol/L calcium, 74 mmol/L chloride, and a pH of 7.53, consistent with hyponatraemia, hypokalaemia, hypochloraemia, and metabolic alkalosis. Her plasma aldosterone level was 137.19 pg/mL (normal range: 10–160 pg/mL), her angiotensin level was 137.67 pg/mL (normal range: 25–129 pg/mL), and her renin activity was 67.17 pg/mL (normal range: 4–24 pg/mL). A urine test revealed a pH of 8.00 and a protein concentration of 1+. Electrolyte levels in her urine and stool were not detected. The patient was administered intravenous fluids and oral electrolyte supplements with an oral chloride intake of 6 mmol/kg/d in the form of NaCl and KCl solutions. With treatment, her serum pH balance and electrolyte levels returned to normal, her urine pH decreased to 6.50 with negative urine protein, and her bowel movements decreased to only 4–5 per day (from 8 to 10 per day), though her stool remained watery.

**Fig. 1:**
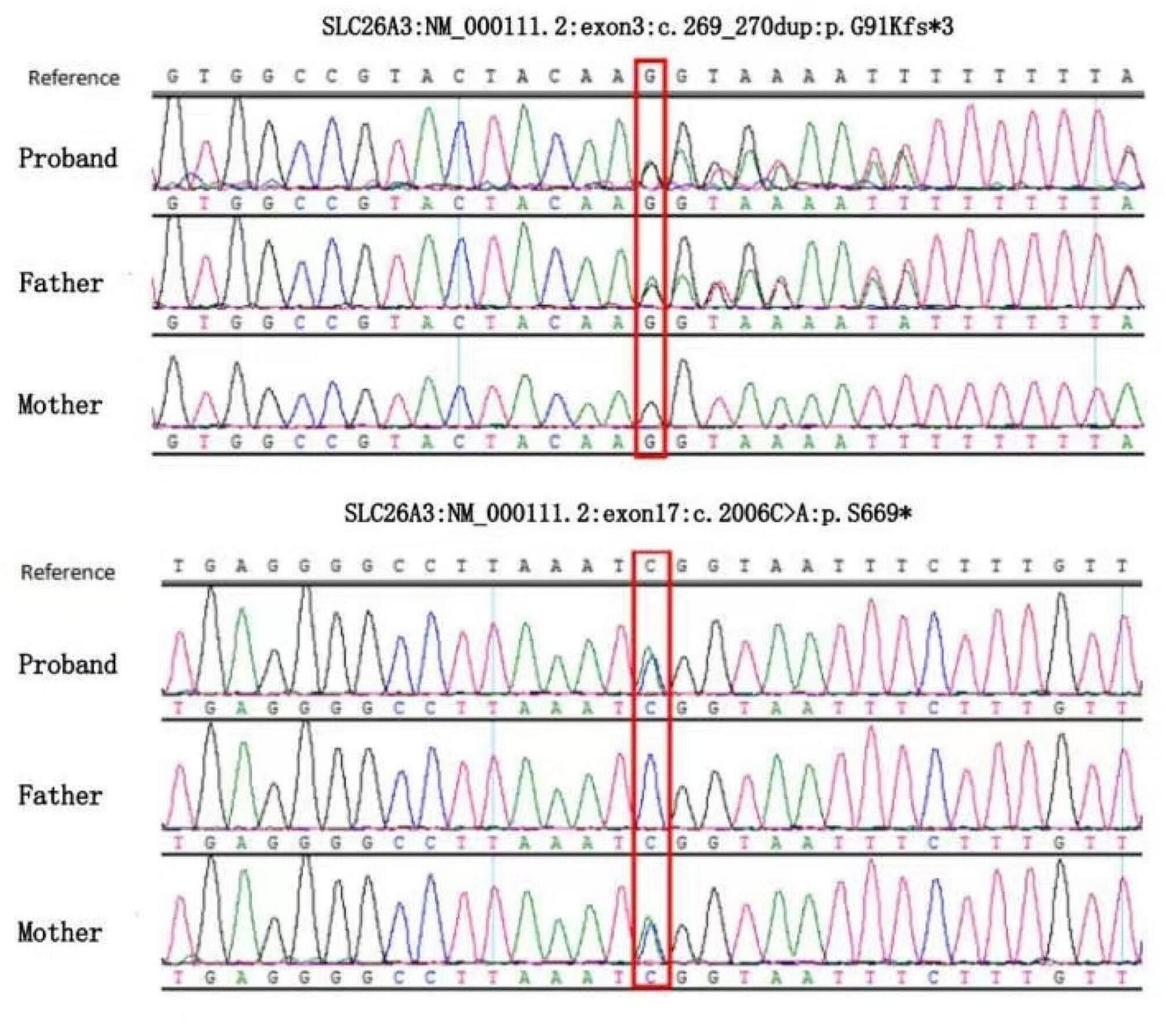
Identification of *SLC26A3* variants. Sanger sequencing confirmed that the compound heterozygous mutations in the *SLC26A3* gene of the proband were inherited from the father and mother

To confirm the suspected clinical diagnosis of CCD, whole-exome capture and sequencing were performed on genomic DNA samples from the patient, and Sanger sequencing was subsequently used to verify candidate mutations in DNA samples from her parents. This analysis confirmed that the patient harboured a compound heterozygous mutation in the *SLC26A3* gene (c.269_270dup: p.G91Kfs*3 and c.2006 C > A:p.S669*, which were inherited from her father and mother, respectively(Fig. 1). Her parents were heterozygous carriers and did not present any symptoms.The frequency of the former in Population Genome Mutation Frequency Database (gnomAD) is 0.000007985; the frequency of the latter in Human Exon Database (ExAC) is 0.0001. According to American College of Medical Genetics and Genomics (ACMG) criteria, the two sites are classified as pathogenic variants (PVS1 + PM2 + PM3).

The patient received regular follow-up with oral sodium and potassium chloride supplements for 4 years since her initial diagnosis. She experienced persistent watery diarrhoea (usually 3–4 times per day, increasing to 5–6 times per day in some cases). However, the child experienced severe electrolyte disorders several times due to poor compliance and a reduction of drug dose, and her condition improved after increasing the dose of drugs. Her delayed growth improved gradually. She had learned to sit without support at 8 months of age and to walk at 15 months of age. She reached 105 cm in height and 19 kg in weight at 56 months of age with normal electrolyte levels and good mental development. The girl was given oral potassium chloride tablets (2.5 g/day) in recent follow-up.

## Discussion

Members of the SLC26 protein family are responsible for transport of small ions, including formate, oxalate, sulfate, Cl^−^, and HCO3^−^. SLC26A3 protein expression is evident throughout the intestinal tract, with maximal levels in the ileum, colon, and duodenum, where it is responsible for Cl^–^/HCO3– exchange, regulating electroneutral NaCl absorption and promoting absorption of water within the small intestine and colon. Mutations that disrupt normal SLC26A3 functionality in humans result in CCD, which can cause severe diarrhoea both *in utero* and postnatally [9]. A diagnosis of CCD can typically be made in children based upon a combination of clinical symptoms, abnormal biochemical findings, and detected genetic mutations. Children with CCD present with early intrauterine symptoms of polyhydramnios, foetal bowel loop dilation, and a lack of meconium. These children are commonly born prematurely, and their excessive watery diarrhoea results in a range of symptoms, including dehydration, hyponatraemia, hypochloraemia, hypokalaemia, and metabolic alkalosis [10]. Often, these children are inaccurately diagnosed with phenotypically similar conditions, such as Bartter’s syndrome [11], which is an autosomal recessive salt-loss tubulopathy that presents with symptoms such as hypokalaemic metabolic alkalosis and normal or low blood pressure despite hyperreninaemia and hyperaldosteronaemia. *SLC26A3* gene sequencing can facilitate definitive diagnosis of CCD.

The patient described in this report exhibited a typical CCD presentation, beginning with intrauterine onset, as evidenced by maternal polyhydramnios. Shortly after birth, she began to exhibit frequent, severe, watery diarrhoea that resulted in dehydration, hyponatraemia, hypokalaemia, hypochloraemia, metabolic alkalosis, failure to thrive, hyperreninaemia, and hyperaldosteronaemia. Genetic testing confirmed that she harboured a compound heterozygous *SLC26A3* mutation (c.269_270dup and c.2006 C > A). This patient appeared to be easily diagnosed, but she was misdiagnosed with infectious diarrhoea or noninfectious diarrhoea (such as lactose intolerance) at a local hospital before admission to our hospital. This kind of congenital diarrhoea is very difficult to correct with routine antidiarrhoeal treatment and lactose-free milk. It is quite difficult to differentiate CCD from infectious disease (such as viral or bacterial infection) or noninfectious diarrhoea, including dietary factors, climate factors, and other congenital diseases, such as Bartter syndrome, congenital sodium diarrhoea, and congenital tufting enteropathy. The reasons for misdiagnosis might include the following. (1) CCD is a rare disease, and clinicians have insufficient understanding of its clinical characteristics and diagnostic criteria, which are not considered in the diagnostic phase of the disease. (2) The clinical manifestations of CCD are similar to those of Bartter syndrome. Compared with CCD, Bartter syndrome is relatively more common; however, severe diarrhoea is absent, and the output of urine potassium and urine chlorine is increased in individuals with Bartter syndrome. (3) Futher investigation was lacking, including urine and stool electrolyte tests, blood tests, immunological studies, endoscopy and biopsies for histology and electron microscopy, and next-generation sequencing (NGS). Indeed, NGS plays an important role in identifying the genetic mutations responsible for congenital diarrhoea and enteropathies [12]. Therefore, more attention should be given to CCD, and further investigations, especially NGS, should be considered when an infant presents with prolonged bouts of severe watery diarrhoea.

The phenotype of CCD seems to be quite consistent globally, and no phenotype–genotype correlations have been reported. To better understand the clinical and genetic characteristics of Chinese children with CCD, we collected data of 14 children (including the present patient) published in Chinese and English within the past 9 years. Among these 14 Chinese CCD patients, 6 were male, 5 female, 3 no exact gender. Ten patients (90.9%, 10/11) presented with intrauterine polyhydramnios, two patients (18.2%, 2/11) exhibited prenatal foetal bowel loop dilation, 8 patients (72.7%, 8/11) were born prematurely, and all 14 patients presented with watery diarrhoea. Dehydration was observed in 10 of these patients (90.9%, 10/11); all 14 exhibited hypokalaemia, hypochloraemia, and metabolic alkalosis. Hyponatraemia was found in 11 patients (100%, 11/11). Hyperreninaemia was evident in 6 patients (85.7%, 6/7), and 5 patients (71.4%, 5/7) presented with hyperaldosteronaemia. Failure to thrive was observed in 8 patients (100%, 8/8). Transient mild proteinuria was detected in 2 patients and nephrocalcinosis in 1 patient. Abnormal renal function was not observed in 11 patients. The incidences of polyhydramnios, foetal bowel loop dilation, preterm delivery, and chronic kidney disease in Chinese patients were lower than those in previous studies [13]. The difference might be related to differences in geographic area and the small sample size.

Ninety-eight variants of the *SLC26A3* gene have been found, including missense, nonsense, insertion, frameshift, deletion and splice variants. The hot spots in Finland, Poland and Arab countries are c.951_953del (p.V318del), c,2024_2026dup (p.I675dup) and c.559G < T(p.G187X), respectively [14]. The c.2063-1G > T variant is the most commonly identified mutation among patients in Japan and Korea [7, 8]. We found 13 variants of the *SLC26A3* gene in 14 Chinese patients with CCD, including 8 missense (c.1631T > A, c.239G > A, c.2048T > A, c.1387 C > T, c.1483 C > A, c.358G > A, c.634G > T, c.1039G > A), 2 nonsense variants (c.1696 C > T, c.2006 C > A), 1 frameshift (c.269_270dup/270_271dup) and 2 splice variants (c.735 + 4_735 + 7del, c.2063-1G > T) (Table 1). Among these mutation sites, the c.269_270dup/270_271dup variant (p.Gly9lLysfs*3) is most common in Chinese patients (50%, 7/14). The population frequency of this variant is 0.000007985 in GnomAD and 0.0001089 in East Asia.

**Table 1:** Clinical and genetic features of Chinese patients with CCD

Items	1	2	3	4	5	6	7	8	9	10	11	12
Authors	Song FY et al [15]	Zhang W et al [16]	Liu YP et al [17]	Lei FY et al [18]	Guo H et al [19]	Wang ZH et al [20]	Li YH et al [21]	Lin LF et al [22]	Sun M et al [23]	Yan W et al [24]	Yin H et al [25]	Li Q (This report)
Sex	male	male	female	female	male	male	female	female	male	male	3, ND	female
Age of onset	36d	3d	2 m	7d	1d	2 m	10d	15d	4 m	3 m	ND	1d
Foetal polyhydramnios	Y	Y	N	Y	Y	Y	Y	Y	Y	Y	ND	Y
Prenatal dilated foetal bowel loops	N	Y	N	N	N	N	N	N	N	Y	ND	N
Premature	Y	Y	N	Y	Y	Y	N	Y	Y	Y	ND	N
Diarrhoea	Y	Y	Y	Y	Y	Y	Y	Y	Y	Y	Y	Y
Dehydration	Y	Y	Y	Y	Y	Y	Y	N	Y	Y	ND	Y
Hypokalaemia	Y	Y	Y	Y	Y	Y	Y	Y	Y	Y	Y	Y
Hyponatraemia	Y	Y	Y	Y	Y	Y	Y	Y	Y	Y	ND	Y
Hypochloraemia	Y	Y	Y	Y	Y	Y	Y	Y	Y	Y	Y	Y
Metabolic alkalosis	Y	Y	Y	Y	Y	Y	Y	Y	Y	Y	Y	Y
Hyperreninaemia	Y	ND	Y	N	Y	ND	ND	Y	Y	ND	ND	Y
Hyperaldosteronaemia	Y	ND	Y	N	Y	ND	ND	Y	N	ND	ND	Y
Failure to thrive	Y	ND	Y	Y	Y	Y	Y	ND	ND	Y	ND	Y
Renal involvement	Small calculi in both renal pelvises	Normal	Normal	Transient mild proteinuria	ND	Normal	Normal	Normal	Normal	ND	ND	Transient mild proteinuria
SLC26A3 variant	c.1631T > A	c.239G > A	c.270_271insAA, c.2048T > A	c.1631T > A	c.1387 C > T, c.1483 C > A	c.270_271insAA	c.358G > A, c.634G > T	c.269 270dupAA, c.735 + 4_735 + 7del	c.269_270dupAA	c.1696 C > T c.269_270dupAA	1 c.1631T > A; 1 ,c.1039G > A, c,2063-1G > T; 1 c.270_271insAA, c.2063-1G > T	c.269_270dup, c.2006 C > A
Protein changes	p.I544N	p.G80D	p.G91Kfs*3, p.I683N	p.I544N	p.R463* p.Q495K	p.Gly9lLysfs*3	p.G120S p.G212C	p.G91Lysfs*3 -	p.G91Lysfs*3	p.R566* p.G91lysfs*3	p.I544N; P.A347T, -, p.G91Lysfs*3, -	p.G91Lysfs*3 p.Ser669Ter
Type of variant	Missense	Missense	Frameshift Missense	Missense	Missense, Missense	Frameshift	Missense, Missense	Frameshift Splicing	Frameshift	Nonsense Frameshift	Missense; Missense, Splicing; Frameshift, Splicing	Frameshift, nonsense
Allele frequency in GnomAD	ND	ND	0.000007985, ND	ND	0.000003986, ND	0.000007985	0.00003183, ND	0.000007985, 0.000007984	0.000007985	0.000003981, 0.000007985	ND; 0.000003983,0.00003618; 0.000007985,0.00003618	0.000007985, ND
Allele frequency in GnomAD-East Asia	ND	ND	0.0001089, ND	ND	0, ND	0.0001089	0.0001087, ND	0.0001089, 0	0.0001089	0, 0.0001089	ND; 0.00005437,0.0004901; 0.0001089,0.0004901	0.0001089, ND

Y: Yes; N: No; ND: No data; GnomAD: Genome Aggregation Database

Early diagnosis and adequate treatment are both critical to facilitate normal physical and mental development in children with CCD. There are currently no specific treatments available for this disorder, and affected individuals must consume sodium and potassium chloride solutions throughout their lives to maintain appropriate electrolyte, acid‒base, and fluid homeostasis. Eleven Chinese patients with CCD were initially treated via the adequate replacement of chloride with sodium and potassium salts administered intravenously and/or orally. A majority of these patients exhibited excellent clinical responses, as evidenced by improvements in mental development and weight gain. The patient in our report received 4 years of treatment and achieved favourable improvement. However, the child experienced severe electrolyte disorders several times due to poor compliance and a reduction of drug dose, and her condition improved after increasing the dose of drugs. Therefore, good compliance and regular follow-up are essential to minimize the risk of long-term complications for CCD patients.

In conclusion, CCD should be considered when an infant presents with prolonged bouts of severe watery diarrhoea, particularly with a history of foetal bowel loop dilation and maternal polyhydramnios. Genetic testing plays a decisive role in its diagnosis and differential diagnosis.

## Data Availability

The original contributions presented in the study are included in the article, and further inquiries can be directed to the corresponding author upon reasonable request.
